# Semantics Based on the Physical Characteristics of Facial Expressions Used to Produce Japanese Vowels

**DOI:** 10.3390/bs10100157

**Published:** 2020-10-13

**Authors:** Shushi Namba, Toshimune Kambara

**Affiliations:** 1 Psychological Process Team, BZP, Robotics Project, RIKEN, 2-2-2 Hikaridai, Seika-cho, Soraku-gun, Kyoto 6190288, Japan; shushi.namba@riken.jp; 2 Department of Psychology, Graduate School of Education, Hiroshima University, 1-1-1 Kagamiyama, Higashi-Hiroshima, Hiroshima 7398524, Japan

**Keywords:** semantic differential scales, facial expressions, sound symbolism, vowels, mouth shape

## Abstract

Previous studies have reported that verbal sounds are associated—non-arbitrarily—with specific meanings (e.g., sound symbolism and onomatopoeia), including visual forms of information such as facial expressions; however, it remains unclear how mouth shapes used to utter each vowel create our semantic impressions. We asked 81 Japanese participants to evaluate mouth shapes associated with five Japanese vowels by using 10 five-item semantic differential scales. The results reveal that the physical characteristics of the facial expressions (mouth shapes) induced specific evaluations. For example, the mouth shape made to voice the vowel “a” was the one with the biggest, widest, and highest facial components compared to other mouth shapes, and people perceived words containing that vowel sound as bigger. The mouth shapes used to pronounce the vowel “i” were perceived as more likable than the other four vowels. These findings indicate that the mouth shapes producing vowels imply specific meanings. Our study provides clues about the meaning of verbal sounds and what the facial expressions in communication represent to the perceiver.

## 1. Introduction

According to a famous Japanese proverb, “names and natures do often agree.” Consistent with this proverb, verbal sounds often agree with specific representations. If people listen to the meaningless sounds of “bouba” or “baluma,” they imagine a circular and expansive shape. When participants judged which figure is “bouba” when presented a pointy figure and a round figure, the most participants selected the round figure as “bouba” [1,2,3]. Vowel sounds are also directly associated with the shape the mouth takes when producing vowels. For example, two previous studies found that the mouth shape made to produce the vowel/a/tends to be bigger than that made to produce a vowel/i/, and that the mouth shapes made to produce words including the vowel/a/also tend to be bigger than those of words including the vowel/i/ [4,5]. Associations between verbal sounds and size concepts are common in some languages [6]. However, the interpretations and observations of these studies only pertain to the oral cavity, tongue position, and acoustic properties of vowels, and not mouth shapes when pronouncing them [4,5,6]. In this study, we aimed to investigate how participants evaluated mouth shapes used to produce vowels by semantic differential scales measuring sensory-motor and emotional imageries.

Facial display is a tool well known to humans, and it facilitates our social communication; in successful communication, perceivers usually try to understand targets’ internal states by focusing on what they are saying as well as what they are expressing through their facial configurations [7]. In fact, facial expressions are a means of conveying a wide range of information [8,9,10,11,12]. While facial expressions commonly provide a clue about which emotions an expresser may be experiencing [13,14], inferences made exclusively from facial movements may not be in accordance with the actual feelings of the expresser, mainly because facial movements can be caused by several factors. Furthermore, Crivelli and Fridlund have criticized the existence of universal “facial expressions of emotion” because there is no consensual scientific definition for “emotion” [15]. Therefore, scholars are now skeptical of the common belief that someone scowls only when expressing anger [16,17]. Recent studies have emphasized how we perceive facial expressions and how we assign them semantic information [18]. Thus, it is important to accumulate evidence about what information perceivers generate and how they produce absent categorical thinking when faced with others’ facial movements.

To understand how perceivers receive information from facial expressions, a specific assumption may be helpful: perceivers make inferences about each facial action separately, not holistically; that is, one’s physical facial action components (e.g., mouth shape) instead of a whole face may have an impact on perceivers’ inferences about others’ facial expressions. Indeed, Durán et al. showed in their meta-analysis on the coherence between facial expressions and emotional experiences that prototypical whole facial patterns were not coherent with the emotional experiences of the expressers, whereas there was a small coherence between their emotional experiences and individual facial components [19]. Moreover, Hyniewska et al. illustrated how each facial component could be associated with the perceivers’ specific decoded emotions [20]. Furthermore, Reschke et al. showed that physical changes of the mouth expressing disgust induced different emotional perceptions [21]; the disgust expression with an open mouth, compared to the disgust expression with a closed mouth, can lead to different inferences according to the type of contextual information available, such as posture-scene stimuli. Additionally, the importance of facial components has been pointed out not only in the decoding process but also in the encoding process. Namba et al. suggested that some facial components were related to the expressers’ self-reported emotional experiences, and these findings supported the understanding that the facial component approach could be informative in figuring out what response each facial action evokes in people [22]. Therefore, highlighting the role of each facial component could lead to more in-depth knowledge about how perceivers receive information from facial movements.

When people pronounce words referring to smallness (e.g., chiisai, in Japanese), these words are usually associated with and pronounced in high acoustic frequency, whereas words referring to largeness (e.g., ookii, in Japanese) are usually associated with low acoustic frequency [6]. Thus, somehow the meaning of words appears to be connected to their physical features. Additionally, verbal sounds are usually directly associated with sensory-motor and emotional experiences (i.e., sound symbolism) [4,5,23]. From the viewpoint of sound symbolism, subjects tend to evaluate a word including the vowel/a/as bigger than that including the vowel/i/ [5]. This evaluation may be associated with the size of the mouth when producing these verbal sounds with different vowels; the mouth gets bigger when producing the vowel/a/than when producing the vowel/i/. Concomitantly, verbal sounds are related to emotional experiences. Rummer et al. identified the relationship between vowel identity and emotional state, in that a subject’s positive mood made them tend to produce more words containing/i:/, and a negative mood made them produce more words containing/o:/ [24]. Supporting this relationship between vowel identity and emotional state, an earlier study on facial feedback showed that facial movements in themselves can result in alteration of affective states [25]. Furthermore, an even earlier study by Zajonc et al. showed that voicing the vowel “ü” might induce negative affective states, while voicing “e” might induce positive affective states [26].

However, these prior studies investigating mouth shapes made to produce vowels failed to evaluate what perceivers infer about mouth shapes when expressers produce all five vowels, so there is little literature available on the evaluation of the perception of each facial component. In social situations such as a casual conversation with another person, we interpret body language and verbal information simultaneously [7], and vowels such as “a,” “i,” “u,” “e” and “o” are usually required. Thus, accumulating evidence about how perceivers make their inferences based on facial components present upon speaking each vowel—apart from the meaning of the word—can be considered as the first step toward a fine-grained examination of how we interpret the meaning behind facial expressions. Such examinations may allow researchers of the Japanese language to further understand the implications of sound-symbolic words with verbal sounds that have specific meanings based on visual information such as mouth shapes. Analyzing how we derive semantic information from the specific components of facial expressions while uttering vowels may be an important way to enhance our knowledge of how people perceive facial expressions.

To elucidate the relationships between specific facial expressions and perceived information, we conducted an exploratory investigation to evaluate what each vowel mouth shape stands for. The current study explored Japanese participants’ responses toward their visual recognition of mouth shapes made by expressers when pronouncing five Japanese vowels (“a,” “i,” “u,” “e” and “o”). We assumed participants would have associative memories between the mouth shapes and the vowels. We focused on the Japanese language and Japanese native speakers as the target language and sample, respectively, because Japanese includes numerous sound-symbolic words, which indicate non-arbitrary associations between verbal sounds and sensory-motor/emotional features [23]. A dictionary of Japanese sound-symbolic words includes more than 4500 sound-symbolic words [27]. Although recent studies have investigated the evaluation, comprehension, and learning of Japanese symbolic words [28,29,30,31,32,33,34,35,36,37], studies associated with Japanese were inconclusive when compared to studies on Western languages [4,5,38,39,40,41,42,43,44].

## 2. Materials and Methods

### 2.1. Participants

Eighty-one Japanese participants (57 women, 23 men, and one gender non-specific person; age *M* = 19.86 years, *SD* = 10.74) with normal or corrected-to-normal vision took part in the current study. Informed consent was obtained from each participant before the investigation. After a lecture on introductory psychology, all participants received a QR code to perform the experimental task voluntarily. All tasks were performed with a smartphone. This study was approved by the Ethics Committee of the Graduate School of Education, Hiroshima University, and was conducted in accordance with the Declaration of Helsinki (approval code: 2019554). Participants did not receive any remuneration for their participation.

### 2.2. Materials

**Figure 1:**
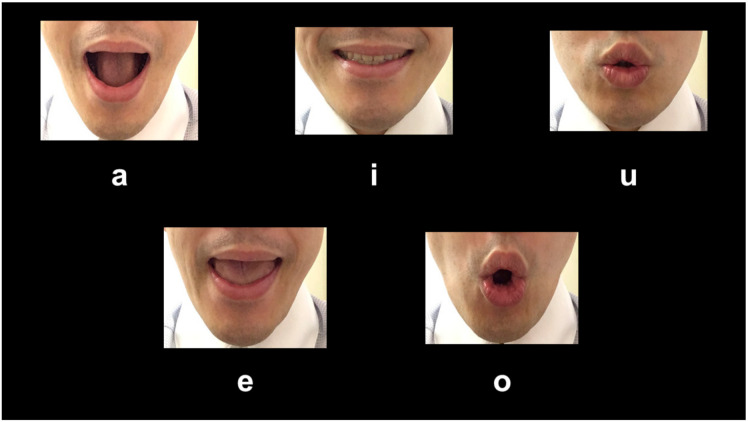
Five mouth shapes studied that voice a Japanese vowel.

The stimuli images consisted of five isolated lower facial parts of a male voicing each vowel: “a,” “i,” “u,” “e” and “o.” Figure 1 represents all five isolated facial parts while pronouncing these vowels. We followed the mouth shapes provided by Hara and Endo in their study [45].

Then, a five-item semantic differential scale [46] was implemented for each target to measure participants’ perception of the mouth shapes using 10 adjectives: size (Q1; 1: small; 5: big), distance (Q2; 1: close; 5: far), thickness (Q3; 1: thin; 5: thick), extent (Q4; 1: narrow; 5: wide), weight (Q5; 1: light; 5: heavy), height (Q6; 1: low; 5: high), depth (Q7; 1: shallow; 5: deep), preference (Q8; 1: hate; 5: like), arousal (Q9; 1: calm; 5: excited), and familiarity (Q10; 1: familiar; 5: unfamiliar). Semantic differential scales have been used for the assessment of visual images [47,48] and Japanese sound-symbolic words [32]. These measurements were chosen on the basis of previous psychological and linguistic studies [23,32,46].

### 2.3. Procedure

Participants were tested individually using Qualtrics software (Provo, UT, USA). All participants were given a link to the experiment so that they were able to perform it during their own personal time. After completing and providing informed consent via Qualtrics, participants were asked to evaluate each image by 10 semantic differential scales, which comprised a block [46]. All five images were evaluated in the same manner. In total, each participant performed 50 trials, and the order of the images was randomized among participants. Furthermore, the 10 adjective semantic differential scales provided during the evaluation task were shown in a randomized order among participants.

### 2.4. Statistical Analysis

To compare evaluations among the lower facial configurations voicing a vowel, we conducted one-way within-subjects ANOVA tests for the effect of the vowels (“a,” “i,” “u,” “e” and “o”). If there was a significant effect, we further analyzed pairwise comparisons using Shaffer’s modified sequentially rejective Bonferroni procedure, and this was performed for all 10 adjectives (Q1–Q10). All analysis was performed by R (3.6.1)

## 3. Results

First, the relationships among adjectives (the dependent variables) were examined. Table 1 describes the correlation among the dependent variables. As shown in Table 1, the results reveal that size and extent had strong positive correlations (*r* = 0.57), while preference and unfamiliarity had negative correlations (*r* = −0.43). The meaning of these pairs appears to be similar, so they make sense when taken together. As for preference and familiarity, the more familiar a shape, the more it will be preferred. Results for each mouth shape are separately reported below.

### 3.1. Big-Small (Q1: Size)

**Figure 2:**
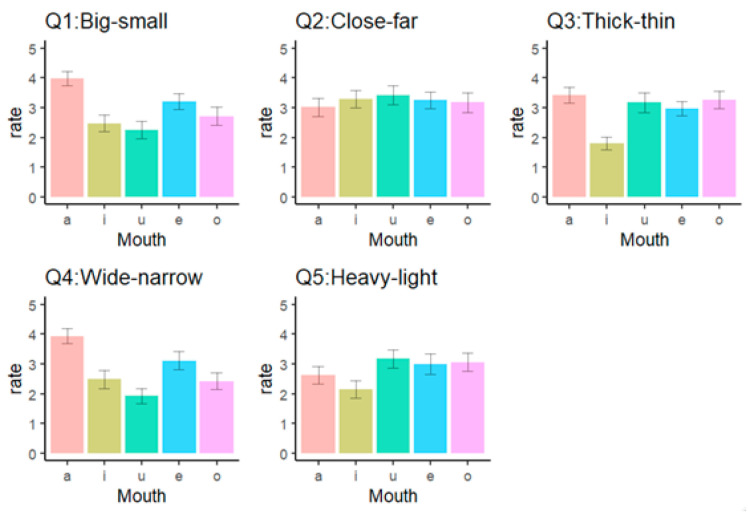
Results of the analysis of Q1 (size), Q2 (distance), Q3 (thickness), Q4 (extent), and Q5 (weight).

Figure 2 describes participants’ judgement of Q1–Q5 for each lower facial configuration voicing a vowel. The results indicate a significant effect of facial configurations (*F*(4, 320) = 25.69, *p* < 0.001, partial *η2* = 0.24). The mouth shape made to voice the vowel “a” was significantly bigger than other configurations (*t*s(80) > 4.92, *p*s < 0.001), and the mouth shape made to voice the vowel “e” was also significantly bigger than the other two vowels: “i” and “u” (*t*s(80) > 4.43, *p*s < 0.002).

### 3.2. Close-Far (Q2: Distance)

The results indicate a significant effect of facial configurations (*F*(4, 320) = 1.21, *p* = 0.31, partial *η2* = 0.02). There was no difference in perception about distance for each facial configuration.

### 3.3. Thick-Thin (Q3: Thickness)

The results indicate a significant effect of facial configurations (*F*(4, 320) = 22.73, *p* < 0.001, partial *η2* = 0.22). The mouth shape made to voice the vowel “i” was significantly thinner than other configurations (*t*s(80) > 6.50, *p*s < 0.001).

### 3.4. Wide-Narrow (Q4: Extent)

The results indicate a significant effect of facial configurations (*F*(4, 320) = 31.86, *p* < 0.001, partial *η2* = 0.29). Consistent with the evaluation of size on Q1, which correlated highly with this item (*r* = 0.54), the mouth shapes made to voice the vowels “a” and “e” were significantly wider than other configurations (*t*s(80) > 2.98, *p*s < 0.02), while “a” was wider than “e” (*t*(80) = 5.13, *p*s < 0.001).

### 3.5. Heavy-Light (Q5: Weight)

The results indicate a significant effect of facial configuration (*F*(4, 320) = 7.97, *p* < 0.001, partial *η2* = 0.09). The mouth shape taken to voice the vowel “i” was significantly lighter than the mouth shapes voicing “u,” “e” or “o” (*t*s(80) > 3.73, *p*s < 0.005).

### 3.6. High-Low (Q6: Height)

**Figure 3:**
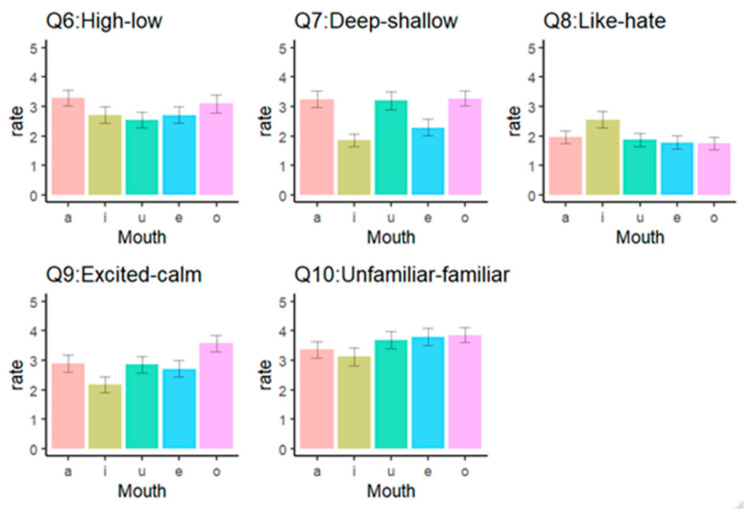
Results of the analysis of Q6 (height), Q7 (depth), Q8 (preference), Q9 (arousal), and Q10 (familiarity).

Figure 3 describes participants’ judgement of Q6-Q10 for each lower facial configuration voicing a vowel. The results indicate a significant effect of facial configurations (*F*(4, 320) = 5.11, *p* < 0.001, partial *η2* = 0.06). The mouth shape made to voice the vowel “a” was significantly higher than the other vowels: “i,” “u” and “e” (*t*s(80) > 3.02, *p*s < 0.02). Additionally, the mouth shape made to voice the vowel “o” was also significantly higher than that of “u” (*t*(80) = 2.83, *p*s = 0.04). Other comparisons were not significant (*p*s = ns).

### 3.7. Deep-Shallow (Q7: Depth)

The results indicate a significant effect of facial configurations (*F*(4, 320) = 24.84, *p* < 0.001, partial *η2* = 0.24). The mouth shapes made to voice the vowel “i” and “e” were shallower than other configurations (*t*s(80) > 4.33, *p*s < 0.001), while “i” was shallower than “e” (*t*(80) = 2.76, *p*s < 0.03).

### 3.8. Like-Hate (Q8: Preference)

The results indicate a significant effect of facial configurations (*F*(4, 320) = 11.26, *p* < 0.001, partial *η2* = 0.12). As Figure 3 indicated, the mouth shape made to voice the vowel “i” was the only mouth shape that was significantly more liked than other configurations (*t*s(80) > 4.28, *p*s < 0.001).

### 3.9. Excited-Calm (Q9: Arousal)

The results indicate a significant effect of facial configurations (*F*(4, 320) = 15.86, *p* < 0.001, partial *η2* = 0.17). The mouth shape made to voice the vowel “o” significantly provided more arousal than other configurations (*t*s(80) > 3.99, *p*s < 0.001). Furthermore, the mouth shape made to voice the vowel “i” significantly provided less arousal than any other configurations (*t*s(80) > 2.75, *p*s < 0.03).

### 3.10. Unfamiliar-Familiar (Q10: Familiarity: A Reverse Item)

The results indicate a significant effect of facial configurations (*F*(4, 320) = 6.52, *p* < 0.001, partial *η2* = 0.08). The mouth shape made to voice the vowel “i” was more familiar than other shapes (*t*s(80) > 2.76, *p*s < 0.05), except for “a” as Figure 3 showed (*t*(80) = 1.31, *p* = 0.77). Additionally, the mouth shape made to voice the vowel “a” was more familiar than the shape made to voice the vowel “o” (*t*(80) = 2.99, *p* = 0.02). Other comparisons were not significant (*p*s = ns).

## 4. Discussion

The results show that participants were able to make several inferences based on mouth shapes, which were in accordance with the physical characteristics expressed by each mouth shape used to utter each vowel; for example, mouth shapes used to voice the vowels “a” and “e” were perceived as big and wide. Indeed, the mouth shapes that people take to pronounce these vowels are commonly bigger and wider than those of other vowels. This is consistent with previous findings of sound symbolic effects for vowels and their meanings [4,5]. Thus, there was a close relationship between the morphological features of the mouth shapes people used to pronounce the vowels and their respective meanings.

The mouth shape made to voice the vowel “a” was usually seen as the one with the biggest, widest, and highest facial components compared to other mouth shapes. This result is consistent with the evaluation of some sound-symbolic words in a previous study (a Japanese sound-symbolic word, KaaN KaaN, means expansiveness and conspicuousness) [49]. We believe this may have been because perceivers made inferences consistent with physical changes that occur in the x- and y-axis of mouths when they make the shape used to pronounce specific vowels. This result might be consistent with previous findings for the circular and expansive shape called “bouba” and “baluma” [1,2,3]. The different dynamics present in small facial movements (u: see Figure 2) and large movements (a: Figure 2) may give the impression that there was a large expansion, so the perceivers’ evaluations from those mouth movements may be in accordance with the visual properties of the mouth shapes. Thus, we showed that the physical characteristics or shapes our mouths take when uttering specific vowels may support the meaning of the verbal sounds and words. In the current study, the mouth shape to pronounce the vowel “a” did not induce distinct psychological impressions such as preference and arousal, while secondary and derivative sound-symbolic effects of “a” could be perceived as “colorfulness and showiness” [49]. This result may suggest that the appearance of the mouth shape to produce the vowel “a,” which requires the mouth to be opened wide, can indirectly contribute to sound-symbolic effects such as showiness. These findings suggest that not only verbal sounds but also mouth shapes that produce verbal sounds individually could include specific meanings associated with physical and psychological features.

The mouth shape made to pronounce the vowel “i” was perceived as thinner, lighter, and shallower than other mouth shapes. Although a previous finding reported that the mouth shape of “i” was the widest compared to other vowels [50], the participants in the current study evaluated “a” and “e” as wider than “i,” “u,” and “o.” These results might mean that the height of the mouth shape affects the perceptual meanings, especially size and width evaluations, of the mouth shapes to pronounce vowels. This speculation was also supported by a strong positive correlation between size and width in this study, and the relationships between perceptual meanings (e.g., size) and vowels (mil vs. mal) in previous studies [5]. Additionally, this mouth shape was related to perceptions of relaxation (i.e., not aroused), likability, and familiarity. Such perceptions were consistent with Japanese sound-symbolic words including the vowel “i” (siiN: silent representation of environment; kirakira, pikapika: preferable and friendly representation of light) [23,49]. These might reveal that the perceptual meanings one attributes to specific facial components are connected to the psychological meaning of the same stimulus. Indeed, the fact that the mouth shape made to voice the vowel “i” was perceived as more likable was consistent with previous findings showing that a positive mood evoked a tendency to utter more novel words containing/i:/ [24]. From the viewpoint of facial feedback [51], the mouth shape used to pronounce the vowel “i” in our study resembled a smile, which might result in a more positive perception of the mouth shape, like the likable and familiar ones we found in our study. However, the mouth shape used to pronounce the vowel “e” in our study also resembled a smile but did not elicit the same perceptions. This inconsistency suggests that, rather than concluding that participants simply derived their perceptions from the resemblance between the mouth shape and positive facial configurations (such as a smile), participants’ psychological meaning for the mouth shape used to pronounce the vowel “i” might be associated with their perception, which relates to the morphological features of the utterance based on sound symbolism. Furthermore, Hamano reported that the vowel “e” in mimetic words was consistently associated with negative qualities such as inappropriateness or vulgarness [49]. Such negative sound-symbolic effects may have canceled out the morphological positive effects of the mouth shape to pronounce the vowel “e.” Thus, the current study might not have found any semantic characteristics for the mouth shape to produce the vowel “e” that could particularly distinguish it from the other vowels. Differences between evaluations of “i” and “e” supported that our findings could induce new impressions connected to physical mouth form voicing “i” rather than the simple interpretation of a smile.

Furthermore, the mouth shape made to pronounce the vowel “o” was perceived as higher and deeper, producing arousal and unfamiliarity in this study. A previous linguistic study identified that the secondary sound symbolism of the vowel “o” was associated with semantic features such as “modest, unremarkable, unnoticeable, shabby, indistinct, or unsophisticated” [49]. Given these two findings, the dark and negative semantic features of sound-symbolic effects, which the vowel “o” has, might induce vigilance in the perceiver when the mouth shape is visually explicit (i.e., when people specifically view the mouth shape to pronounce the vowel “o”). As a result, the visual information of the mouth shape to pronounce the vowel “o” may cause high arousal and unfamiliarity. This mouth shape can also be seen when people feel surprised [52]. In addition, the vowel “o” in sound-symbolic words represents inconspicuousness [23], and its sound symbolism might contribute to the impression of unfamiliarity from the mouth shape made to pronounce the vowel. These are highly speculative inferences which may be of interest to future research.

### Limitations

This study provided relevant evidence, yet there were several limitations. First, the five-item semantic differential scales assessed seven physical and only three psychological features (preference, arousal, and familiarity); thus, it provided far less information on the psychological state of the sample when compared to several other psychological scales used to assess psychological representations. Therefore, additional studies should be conducted using diverse rating scales, including those with constructs such as competence, attraction, and disgust.

Second, it could be clearly seen in the visual stimuli that there was only one mouth used in the examples, and it was a man’s. Thus, it is difficult to generalize our results, and future studies need to identify these same evaluations regarding stimuli using mouths with female and non-binary gender markers. Additionally, the results of this study were based on samples of Japanese native speakers. Thus, future data collection from samples of other populational groups or remote cultures could prove very meaningful for facial expression research, which could corroborate our research and increase the relevance of our findings.

Third, although our findings are consistent with relationships between words with vowels including high second formants and size [4,5,6], we did not consider a few exceptions [6]. Ohala concretely indicated that there are some exceptions. For example, “small” in English includes vowels of low second formants that commonly represent big meanings (e.g., a Greek word/makros), whereas “big” in English involves vowels of high second formants that commonly represent small meanings (e.g., a Greek word/mikros). Future studies might need to examine why a few alternative exceptions could occur.

Finally, although we examined the subjective evaluations of the mouth shapes to pronounce vowels, the actual words included multiple vowels. In Japanese sound-symbolic words, a linguist suggests that the functions of the first vowels are different from those of the second vowels [23]. For instance, the first vowels represent the initial shape of objects and events, while the second vowels represent the resultant shape of objects and events [23]. Taken together, future studies might need to investigate the combinatorial effects of mouth shapes to pronounce multiple vowels.

## 5. Conclusions

The results show that participants made several inferences in accordance with the physical characteristics of each mouth shape taken to utter each vowel. This was consistent with previous findings of sound-symbolic effects related to vowels and their meanings [4,5]. Furthermore, the results in this study showed that the mouth shapes producing vowels have specific associations; therefore, this study can provide clues about the “meaning” of meaningless words and what the facial expressions in communication represent to the perceiver.

## Figures and Tables

**Table 1:** Correlation matrix among all adjective evaluations.

	Size	Distance	Thickness	Extent	Weight	Height	Depth	Preference	Unfamiliarity
Distance	−0.11								
Thickness	0.30	0.03							
Extent	0.57	−0.01	0.22						
Weight	0.12	0.18	0.36	0.06					
Height	0.16	−0.04	−0.02	0.17	−0.13				
Depth	0.25	0.11	0.34	0.12	0.28	0.06			
Preference	0.00	−0.03	−0.18	0.07	−0.16	0.04	−0.09		
Arousal	0.10	0.08	0.24	0.01	0.19	0.02	0.23	−0.18	
Unfamiliarity	−0.03	0.04	0.13	−0.12	0.23	−0.07	0.08	−0.43	0.23

## Data Availability

The datasets generated for this study are available on request to the corresponding author.
